# Beyond Academics: A Model for Simultaneously Advancing Campus-Based Supports for Learning Disabilities, STEM Students’ Skills for Self-Regulation, and Mentors’ Knowledge for Co-regulating and Guiding

**DOI:** 10.3389/fpsyg.2018.01466

**Published:** 2018-08-17

**Authors:** Consuelo M. Kreider, Sharon Medina, Mei-Fang Lan, Chang-Yu Wu, Susan S. Percival, Charles E. Byrd, Anthony Delislie, Donna Schoenfelder, William C. Mann

**Affiliations:** ^1^Department of Occupational Therapy, University of Florida, Gainesville, FL, United States; ^2^Counseling and Wellness Center, University of Florida, Gainesville, FL, United States; ^3^Department of Environmental Engineering Sciences, University of Florida, Gainesville, FL, United States; ^4^Food Science and Human Nutrition Department, University of Florida, Gainesville, FL, United States; ^5^Department of Community Health and Family Medicine, University of Florida, Gainesville, FL, United States; ^6^Center for Independent Living of North Central Florida, Gainesville, FL, United States

**Keywords:** learning disorders, social support, mentors, self-management, self-regulation, young adult, campus community integration, school culture

## Abstract

Learning disabilities are highly prevalent on college campuses, yet students with learning disabilities graduate at lower rates than those without disabilities. Academic and psychosocial supports are essential for overcoming challenges and for improving postsecondary educational opportunities for students with learning disabilities. A holistic, multi-level model of campus-based supports was established to facilitate culture and practice changes at the institutional level, while concurrently bolstering mentors’ abilities to provide learning disability-knowledgeable support, and simultaneously creating opportunities for students’ personal and interpersonal development. Mixed methods were used to investigate implementation of coordinated personal, interpersonal, and institutional level supports for undergraduate science, technology, engineering, and math (STEM) students with learning disabilities. A one-group pre-test post-test strategy was used to examine undergraduate outcomes. Participants included 52 STEM undergraduates with learning disabilities, 57 STEM graduate student mentors, 34 STEM faculty mentors, and 34 university administrators and personnel as members of a university-wide council. Enrolled for 2 years, undergraduates were engaged in group meetings involving psychoeducation and reflective discussions, development of self-advocacy projects, and individual mentorship. Undergraduates reported improved self-efficacy (*p* = 0.001), campus connection (*p* < 0.001), professional development (*p* ≤ 0.002), and self-advocacy (*p* < 0.001) after two academic years. Graduate student mentors increased their understanding about learning disabilities and used their understanding to support both their mentees and other students they worked with. Council members identified and created opportunities for delivering learning disability-related trainings to faculty, mentors and advisors on campus, and for enhancing coordination of student services related to learning and related disorders. Disability-focused activities became integrated in broader campus activities regarding diversity. This research explicates a role that college campuses can play in fostering the wellbeing and the academic and career development of its students with developmental learning and related disorders. It offers an empirically tested campus-based model that is multilevel, holistic, and strengths-based for supporting positive outcomes of young people with learning disabilities in STEM. Moreover, findings advance the knowledge of supports and skills that are important for self-regulating and navigating complex and multi-faceted disability-related challenges within both the postsecondary educational environment and the young adults’ sociocultural context.

## Introduction

One category of highly prevalent developmental psychopathologies within educational settings is developmental learning disorders. The term learning disabilities (LDs), or specific learning disabilities, as it is often referred to within educational settings, refers to developmental neuropsychological disorders whose primary difficulties are manifested in the areas of reading, writing, and/or mathematics (American Psychiatric Association, 2013; Scanlon, 2013) but also impact multiple areas of life (Sharfi and Rosenblum, 2014). LDs are highly prevalent on college campuses (Newman et al., 2011) and can pose significant challenges for students with these conditions (Cortiella and Horowitz, 2014). These students enroll in college at rates equal to the general population; however, they graduate at lower rates (38–41%) than those without disabilities (52%) (Lorig and Holman, 2003; Cortiella and Horowitz, 2014; Showers and Kinsman, 2017). College students with disabilities have access to a range of both academic (e.g., writing centers) and disability-related supports (e.g., campus disability support office). However, supports for other aspects of a college student’s life, such as social and emotional supports, are also important for bolstering students’ abilities to overcome disability-related challenges (Kreider et al., 2015). Moreover, because the range of needed supports and campus-based resources are not typically provided in a coordinated fashion, students often have to seek out and navigate essential supports and campus resources by themselves.

While core manifestations of LDs primarily impact learning, psychosocial problems are also reported (Tanner, 2009; Sharfi and Rosenblum, 2014). Differences in information processing are regarded to be at the core of LDs (Johnson et al., 2010; Scanlon, 2013), which in turn impacts abilities for efficient and/or full participation in various life domains such as interpersonal interactions and daily life activities (Sharfi and Rosenblum, 2014). Cognitive profiles of individuals with LDs often include difficulties with processing speed, working memory, and/or mental flexibility (Hain et al., 2009). Executive function difficulties in LD can interfere with the higher-level skills of organization, abstract reasoning, attention, planning, and/or time management (Moll et al., 2016; Smith-Spark et al., 2016). Such cognitive processing issues pose additional challenges for students with LDs in science, technology, engineering, and math (STEM) fields of study (Asghar et al., 2017). The inquiry-oriented problem-based learning used in STEM instruction places substantial demands on students’ cognitive processing and speed (Brigham et al., 2011), which can magnify challenges for students with LDs (Asghar et al., 2017). For undergraduates, the 48% STEM-attrition rate (Chen and Ho, 2012) may further compound concerns regarding college persistence for students with LDs in STEM.

Difficulties stemming from LDs can undermine college success, which requires both adequate academic strategies and life skills such as those needed to function within social, interpersonal, self-care, and work contexts (Kim et al., 2010). Examples of life skills that are essential for all college students include productive time utilization (Macan et al., 1990; Lahmers and Zulauf, 2000), strategic organization (VanZile-Tamsen, 2001), and the management of stress and emotional health (Pritchard and Wilson, 2003). However, these essential skills can pose significant challenges for college students with LDs because of associated cognitive processing difficulties. Moreover, these students require additional disability-related skills that include learning to identify needed disability-related supports and seeking help when necessary (Trainin and Swanson, 2005; Kreider et al., 2015). Within the classroom, help-seeking has been shown to be an important strategy for college students with LDs in compensating for cognitive processing difficulties (Trainin and Swanson, 2005).

Effective self-regulation is a critical skill for youth as they transition to adult roles and contexts and engage in the college process (Murray et al., 2015). Self-regulation refers to the cognitive and emotional processes needed to maintain goal directed behavior in the presence of a challenge or adversity. As such, self-regulation requires effective problem solving, goal setting, flexibility, planning, and decision making. However, due to the nature of disability-related cognitive processing difficulties, youth with LDs can face disability-related challenges to the development of adequate self-regulation abilities needed while in college. College students, as emerging adults, are expected to navigate increasingly complex life situations. Successful management of the increasingly complex life situations of late adolescence and young adulthood requires adequate abilities for prioritization, task management, and the setting of realistic goals (Murray et al., 2015). Co-regulation refers to the process used by supportive others that fosters the self-regulation abilities of the young person (Murray and Rosanbalm, 2017). Availability of supportive others who can serve as co-regulators is integral to development of strong self-regulation (Murray et al., 2016).

**FIGURE 1:**
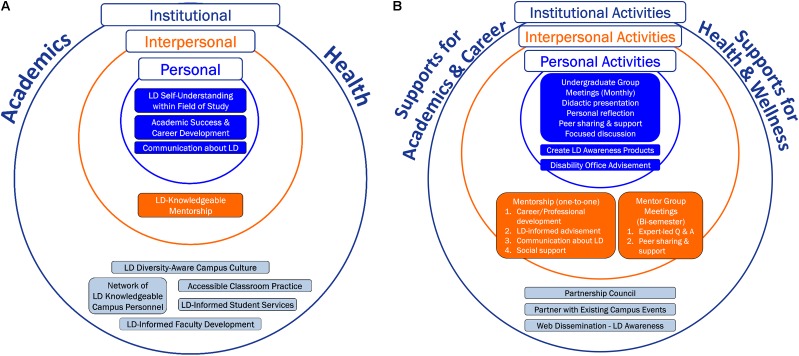
Comprehensive Support for STEM Students with Learning Disabilities (CS^3^LD) Model. **(A)** Conceptual model and **(B)** Implementation model.

To support the academic and psychosocial needs of undergraduates with LDs, we developed a holistic and multi-level model of campus-based supports referred to as the Comprehensive Support for STEM Students with Learning Disabilities (CS^3^LD). The CS^3^LD model is a campus-based framework of integrated interventions that target changes at the institutional, personal, and interpersonal levels. Changes at the institutional level are facilitated through the creation of a campus network of LD-knowledgeable personnel, while interventions for supporting students’ personal and interpersonal development are concurrently offered. Academic supports include an emphasis on career exploration and mentorship, while psychosocial supports are designed to bolster health and wellbeing. The CS^3^LD model is depicted in **Figure 1**; 1A illustrates the CS^3^LD conceptual model and 1B shows activities used for implementing the model.

Personal level supports are aimed at fostering the undergraduate’s self-awareness and understanding about LDs with an emphasis on identifying and advocating for disability-related strengths. Interpersonal level supports center on learning disability-informed mentorship by a graduate student to cultivate professional enculturation to the student’s chosen field of study, with supplementary support for the mentorship from a STEM faculty mentor. Additionally, mentors are assisted in increasing their understanding about LDs. Mentors are also guided in implementation of principles of universal design for learning within their teaching and mentorship. Universal design for learning is an instructional practice that proactively strives to meet the diverse learning needs, strengths, and preferences of all the students who are qualified to enroll in college and the college courses (Burgstahler, 2008). A key institutional level strategy is the creation of a disability-focused Council, referred to as the Partnership Council, to advance a learning disability-aware campus culture.

Integral to the CS^3^LD model is the acknowledgment of the interrelated role of the person and the environment in shaping individuals’ participation and performance, such as performance of academic, social, and health behaviors within their everyday lives (Bandura, 1986, 1998, 2005; Baum and Law, 1997; World Health Organization, 2007; Moll et al., 2016). The CS^3^LD model posits that a student’s knowledge and beliefs, together with the university environment, influence participation and behaviors necessary for the student’s academic success and overall health and wellbeing. We focused this initial implementation and testing of the CS^3^LD model on STEM undergraduates, which was prompted by national prioritization for increasing the number of college graduates who are prepared for the STEM workforce (Chen, 2013). This paper presents the CS^3^LD model, its implementation, the outcomes at each level, and its implications for practice and future research.

## Materials and Methods

### Design

Mixed-methods were used to assess implementation of the CS^3^LD model during the 4-year timeframe of August 2013 through May 2017. Qualitative data from focused group discussions, participant communications, and individual interviews were combined with quantitative data from outcome surveys and implementation data (e.g., recruitment and project activity records) to describe and understand factors influencing: (1) model implementation, (2) implementation outcomes, and (3) potential for institutional adoption of model activities.

**FIGURE 2:**
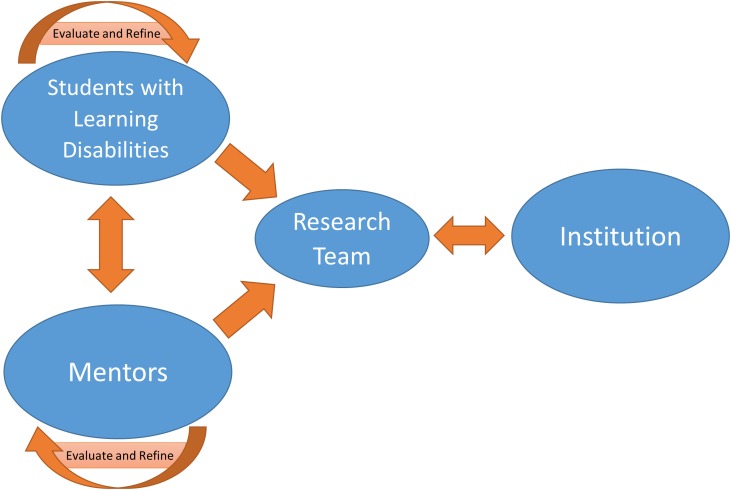
Implementation framework using a continuous improvement research approach.

A continuous improvement process of action and analysis was used to refine implementation of model activities at each level (**Figure 2**), which enabled ongoing evaluation of factors affecting model implementation and outcomes (Peters et al., 2013). Feedback was continuously sought from participating undergraduates, mentors, and our institutional level partners to improve CS^3^LD activities. This process guided steps taken within the same level of the model as well as refinement of activities at the other levels. The continuous improvement process informed shifts in psychoeducational topical content and emphasis of facilitated discussions with undergraduate participants. The process also enabled us to bring information learned from the undergraduates and mentors to institutional level participants for potential campus-level actions.

A participatory action research approach was used as part of the continuous improvement process to facilitate desired culture changes within the university. Participatory action research entails the active engagement of research participants in the research process with an expressed intent for the actions taken to work toward creating social changes (MacDonald, 2012). Undergraduates created disability-awareness projects, which were designed as content for development of public messaging materials for raising awareness about LDs. Mentors engaged in active learning about LDs and the practices for supporting students with diverse learning styles. Campus partners (i.e., Partnership Council) interpreted insights from the undergraduate participants and the mentors to identify potential solutions for improving relevant campus practices.

A one-group pre-test post-test approach was used to examine undergraduate outcomes.

### Subjects and Setting

This study was conducted at the University of Florida, a large research-intensive university in the United States. The study protocol was approved by the University’s Health Science Center Institutional Review Board. All personal level (i.e., undergraduates) and interpersonal level (i.e., graduate students and faculty mentors) participants gave written informed consent. Informed oral consent was obtained from institutional level (i.e., Partnership Council) participants at the beginning of each Partnership Council meeting. Use of oral consent for institutional level participants was approved by the University’s ethics committee. No sensitive information was collected from Council participants or discussed at the meetings, and the composition of attendees at each Council meeting varied based on Council members’ availability.

Personal-level research participants were 52 undergraduate STEM students who were registered with the campus disability office and eligible to receive academic accommodations related to LDs. Participants were deemed eligible based on the campus disability office’s categorization of LDs, which included disorders related to reading, writing, math, coordination, auditory processing, language processing, and/or attention. Undergraduate participants were not excluded if they had a co-morbid mental health condition. Resultantly, 30% (*n* = 16) of the study sample reported a mental health diagnosis other than an attention disorder. Mental health conditions included anxiety, depression, and autism spectrum disorder. The primary source of undergraduate recruitment was the campus disability office, which generated LDs specific listservs, shared study information during new student orientations, and posted handouts and recruitment flyers in the disability office and around campus. We used the National Science Foundation’s definition of STEM, which also includes the social, behavioral, and economic sciences (National Science, and Board, 2014). Undergraduate participants were enrolled for 2 years (i.e., four consecutive non-summer semesters).

Interpersonal-level research participants included 34 faculty and 57 graduate students, who were recruited as mentors for the undergraduates enrolled in this study. Eligible faculty and graduate student mentors were matched with an undergraduate based on the undergraduate’s field of study. Mentors were not expected to have specific knowledge about LDs or disability. Recruitment strategies for faculty and graduate student mentors included word of mouth and campus/department listservs for emailing study advertisements. The campus-wide graduate student listserv was used to recruit our initial wave of graduate student mentors. This recruitment strategy resulted in a large enough pool of potential graduate student mentors, so that subsequent graduate student recruitment only required use of department listservs to target mentors from specific fields of study. Undergraduate and graduate student participants were compensated for each semester that they actively participated in the research activities. **Table 1** reports demographic characteristics of undergraduates with LDs and graduate student and faculty mentors. **Table 2** reports undergraduate participants’ diagnostic and symptom characteristics and areas of difficulties.

**Table 1:** Undergraduate student, graduate student, and faculty mentor characteristics.

Participants [*n*]	Gender *n* (%)	Race *n* (%)	Ethnicity *n* (%)	Mean age (SD)	Field of study *n* (%)
Undergraduates with LD [52]	Male 26 (50) Female 24 (46) Not reported 2 (4)	White 37 (71) Black 8 (15) Asian 1 (2) Other 4 (8) Not reported 2 (4)	Hispanic 9 (17) Non-Hispanic 26 (50) Not reported 17 (33)	21.2 (3.5)^∗^	Physical/Biological sciences☼ 20 (38) Social/Behavioral/ Economic Sciences 14 (27) Technology 3 (6) Engineering 12 (23) Mathematics 3 (6)
Graduate student mentors [57]	Male 28 (49) Female 28 (49) Not reported 1 (2)	White 29 (50) Black 6 (11) Asian 13 (22) Other 3 (5) Not reported 6 (11)	Hispanic 5 (9) Non-Hispanic 39 (68) Not reported 13 (23)	24.5 (4.7)^†^	Physical/Biological Sciences 22 (38) Social/Behavioral/ Economic Sciences 17 (30) Technology 1 (2) Engineering 15 (26) Mathematics 2 (4)
Faculty mentors [34]	Male 19 (56) Female 8 (23) Not reported 8 (21)	White 18 (53) Black 1 (3) Asian 5 (15) Indian 1 (3) Other 2 (6) Not reported 7 (20)	Hispanic 2 (6) Non-Hispanic 31 (91) Not reported 1 (3)	— ^¶^	Physical/Biological Sciences 11 (32) Social/Behavioral/ Economic Sciences 11 (32) Technology 2 (6) Engineering 8 (24) Mathematics 2 (6)

^∗^*n = 51; ☼includes chemistry, physics, astronomy, earth/ocean/atmospheric, agricultural, environmental, life, health sciences; ^†^*n* = 46; ^¶^data not collected*.

**Table 2:** Undergraduate ratings of challenges associated with learning disorders.

Area of difficulty	Overall median rating^∗^ (IQR^†^) *n* = 52	LD only median rating (IQR) *n = 22*	AD only median rating (IQR) *n* = 18	LD/AD median rating (IQR) *n* = 12
Staying focused	75 (62, 94)	63 (49, 85)	90 (73, 98)	75 (67, 89)
Managing time	65 (50, 81)	60 (42, 80)	73 (63, 88)	65 (50, 100)
Extensive writing assignments	65 (31, 85)	58 (26, 84)	65 (31, 75)	80 (56, 89)
Reading comprehension – academic publications	64 (50, 81)	62 (15, 88)	59 (36, 73)	74 (60, 89)
Organization	62 (47, 79)	54 (35, 66)	76 (65, 87)	55 (25, 97)
Memorizing or retrieving information from memory	57 (23, 85)	35 (10, 68)	66 (52, 85)	78 (6, 89)
Following multi-step directions	56 (34, 70)	54 (25, 70)	56 (38, 69)	68 (40, 80)
Expressing thoughts or opinions clearly	52 (22, 71)	40 (13, 70)	53 (23, 60)	68 (57, 79)
Following others speak in conversation	50 (21, 73)	46 (12, 73)	59 (28, 73)	37 (15, 60)
Applying different approaches to one problem	38 (18, 56)	36 (10, 68)	29 (12, 59)	50 (18, 66)
Initiating activities, tasks, or independent ideas	34 (18, 63)	21.5 (9, 50)	52 (30, 70)	34 (5, 56)

^∗^*Digital visual analog scale, 0 = no difficulty, 100 = extreme/constant difficulty, ^†^IQR**=** Interquartile range*.

At the institutional level, we recruited university administrators and personnel (*n* = 34) from campus academic and student service units to serve on the LD-focused Partnership Council. The interdisciplinary investigative team and the director of the campus disability office used their campus networks to facilitate recruitment of participants for the Council via an introductory email inviting participation. Partnership council meetings were held once each semester (i.e., two to three times each year) at a central location on campus. University personnel (i.e., faculty mentors and Partnership Council members) participated in the study without compensation. **Table 3** details the composition of the Partnership Council.

**Table 3:** Composition of CS^3^LD Partnership Council.

Participating entities (*n*^∗^)	Representative type *n* (%)
University Offices: Office of the Provost (2), Office of the Dean of Students (1), Office of Faculty Development and Teaching (2) Colleges and Schools: Education (2), Engineering (3^α^), Health and Human Performance (2^α^), Agricultural and Life Sciences (2^α^), Public Health and Health Professions (2^α^), Liberal Arts and Sciences (1), Graduate School (1) University Centers: Disability Resource Center (2), Teaching Center (1), Counseling and Wellness Center (3^α^), GatorWell (3), Career Resource Center (2), International Center (1), Center for Undergraduate Research (1) Academic Programs: Special Education (2), Howard Hughes Medical Institute Science for Life (1) Student Groups: Student Government Disability Affairs Cabinet (4), Gators for All Abilities (2) Personnel Groups: Academic Advisors Council (1)	Administrator^¶^ 23 (67) Non-administrative faculty 2 (6) Non-administrative staff 5 (15) Undergraduate student leader 4 (12)

^∗^*Some representative members represented two entities and are accounted for in both entities. ^α^Project investigator included in the count. ^¶^Administrator = Dean/Assistant Dean, Department Chair, Program Director*.

### Data Sources

Qualitative data included meeting notes, written feedback sheets, and audio-recordings taken during group meetings with undergraduates, mentors, and the Partnership Council. Focused discussions specific to evaluation of the model’s activities and implementation procedures were held at the end of each academic year. Audio-recordings were transcribed for use in analyses. Instruments used to quantify potential impacts on undergraduate students with LDs included use of the Academic Behavioral Confidence Scale (ABCS) (Sander and Sanders, 2003), the Institutional Integration Scale (IIS) (Pascarella and Terenzini, 1980), the Personal Growth Initiative Scale (PGIS) (Robitschek, 1998), and survey questions developed for this study, which are delineated in **Tables 3**, **4**. Survey questions were assessed to determine face validity and checked for errors such as the presence of double barreled questions and confusing or leading questions. Quantitative instruments were administered at baseline and at the end of each semester for four semesters (i.e., two academic years).

**Table 4:** Topics included in 30 undergraduate group meetings held over four academic years.

Topic	Description	Number (%) of meetings the topic was included^∗^
Communication	Information and strategies about how to communicate with people within students’ lives	16 (29%)
Advocacy	Information about promoting knowledge about LD^∗∗^ or contributing to a more disability friendly environment	15 (27%)
Stress	Information about how stress presents and strategies for managing stressors	13 (23%)
LD-ADHD^†^ differences	LD/ADHD symptoms, cognitive styles, LD/ADHD brain structure and functioning differences, and strategies for highlighting LD/ADHD strengths	13 (23%)
Misconceptions and stigma	Things they wish others understood, and strategies for promoting understanding and coping with stigma	12 (21%)
Time management	Information and strategies about managing time and tasks	11 (20%)
Strengths and challenges	Knowledge about strengths and challenges, and strategies for highlighting strengths	11 (20%)
LD symptom awareness	Understanding/awareness of how LD impacts life and strategies for preventing LD-related problems	11 (20%)
Accommodations	Information and strategies about how to access academic accommodations	10 (18%)
Health behaviors	Information and strategies about healthy behaviors and developing heath promoting daily routines	9 (16%)
Executive functioning in real life contexts	Information about cognitive processes involved in goal directed behavior within students’ everyday life situations and strategies for situational appraisal, prioritization, planning and problem solving within everyday life situations	7 (13%)
Health literacy	Information about general health concepts and strategies for locating and appraising health information	7 (13%)
Self-efficacy	Information and discussions regarding personal judgments in one’s abilities to reach goals	6 (11%)
Anticipatory guidance from guests	Guest speakers with LD/ADHD with an established STEM career sharing their experiences and personal insights	4 (7%)
University resources	Information about available university resources	4 (7%)
Disability in the workforce	Information and strategies for how to get accommodations, engage in interviews as an individuals with an invisible disability, and information regarding how disability accommodations in the workplace are similar and different from classroom accommodations	4 (7%)
Mentorship	Reflection/discussions regarding mentorship impacts on college experiences and/or perspectives	4 (7%)
Imparting wisdom for other students	Reflection/discussions regarding information incoming students should know	2 (4%)
Assistive technology	Information about types of assistive technology	1 (2%)
Relationships	Information about interpersonal and professional communication for developing and maintaining relationships	1 (2%)

^∗^*Included via presentation slides and/or discussion prompts/probes. ^∗∗^LD = Learning disorder. ^†^ADHD = Attention deficit hyperactivity disorder*.

The ABCS is a 24-item self-report questionnaire with adequate reliability and validity in measuring college students’ confidence in performing academically related behaviors (Sander and Sanders, 2003, 2006). Each item is scored on a 5-point scale with the rating of one anchored with *Not at All Confident* and five anchored with *Very Confident*. Higher scores indicate better academic self-confidence. The IIS includes 30 self-report items with adequate reliability and validity in assessing the constructs of academic and social integration of college students (Pascarella and Terenzini, 1980). Items are scored on a 5-point scale (5 = *Strongly Agree*, 4 = *Agree*, 3 = *Not Sure*, 2 = *Disagree*, 1 = *Strongly Disagree*) with 10 items requiring reverse scoring. Higher scores indicate better integration. The PGIS is a 9-item self-report instrument rated on a six point scale (1 = *Strongly Disagree* through 6 = *Strongly Agree*). The PGIS has adequate reliability and validity in assessing a person’s active and intentional engagement in the process of personal growth during a transitional time period (Robitschek, 1998). Higher scores indicate greater engagement in the personal growth process.

### Analysis

Qualitative data were assessed for themes specific to participants’ experiences in order to identify salient and actionable aspects of the CS^3^LD model implementation. An iterative process of ongoing data collection and analysis was used whereby emergent understandings guided subsequent actions and data collections (Richards and Morse, 2013; Richards, 2015). Data were checked immediately for accuracy and completeness, searched for potentially actionable items, and regularly discussed. Decisions regarding refinements to the CS^3^LD model and/or implementation activities were shared with research participants during the group meetings in order to: (1) verify the accuracy of interpretations of the qualitative data, (2) identify potential barriers to implementation of new or modified activities, and (3) ensure the acceptability of any modification to the activities and/or the model.

Descriptive statistics were used to assess demographic and symptom variables and survey item responses regarding perceived CS^3^LD impacts. Quantitative data were checked for normality using Shapiro-Wilk tests and visual inspection (e.g., histogram). Resultantly, non-parametric statistics (i.e., Wilcoxon signed-rank tests) were used to test within-subjects differences in the undergraduates’ ABCS scores, IIS scores, PGIS scores, STEM professional development questionnaire ratings, and self-advocacy questionnaire ratings at baseline and after four semesters (i.e., 2 years). Analyses were run using IBM SPSS Statistics for Windows (Version 24.0, NY: IBM Corp) with two-tailed significance set at the more conservative α = 0.01 due to the multiple measures tested.

## Results

### Model Implementation

#### Personal Level Implementation

Undergraduate group meetings were held monthly during the fall and spring semesters and were led by the study investigators with expertise in disability and LDs. During these group meetings, topical content was provided and followed by focused discussions regarding the students’ disability-related experiences specific to the topic. Focused discussions also included strategy sharing, strategy refinement, problem solving for coping with challenges, and problem solving and/or strategizing for preventing future anticipated challenges. Topics were initially informed by the literature and selected from the domains of academics, career, health, and wellness. Undergraduates provided on-going feedback as to the topics selected and the development and refinement of new topics and focused discussions. Topics from the academic and career domains included communication with professors, academic accommodations, assistive technology, and transition to the workplace. Topics from the health and wellness domains included cognitive styles, stress and time management, and communication about LDs to friends and family. Group discussions and participant feedback repeatedly indicated that, although learning and attention disorders are lifelong developmental conditions primarily diagnosed through the health system, our undergraduates with learning disabilities did not view themselves as having a health concern but rather a learning difference. This impacted the content of psychoeducational topics presented within the undergraduate group meetings. Resultantly, health-related topics and group discussions focused on strategies and resources for managing stress and supporting overall wellbeing instead of healthful behaviors, health literacy, or accessing adult system health services. **Table 4** details topics included during monthly undergraduate group meetings. Undergraduate group meetings were held on campus with opportunities offered for making up missed meetings; the overall attendance rate was 85%.

**FIGURE 3:**
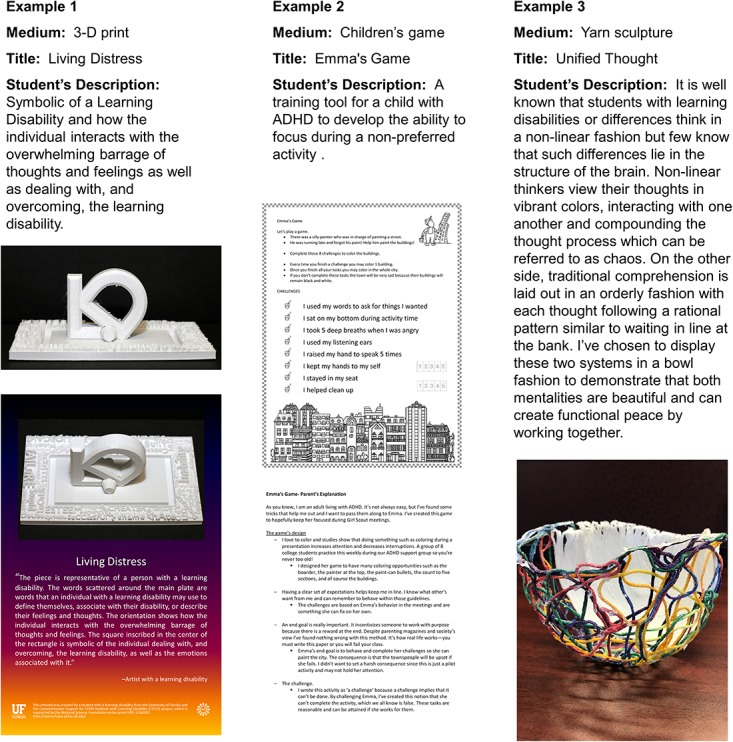
Examples of disability advocacy/awareness project mediums, titles, descriptions, and projects as prepared by the student and/or as disseminated as part of institutional level activities.

**FIGURE 4:**
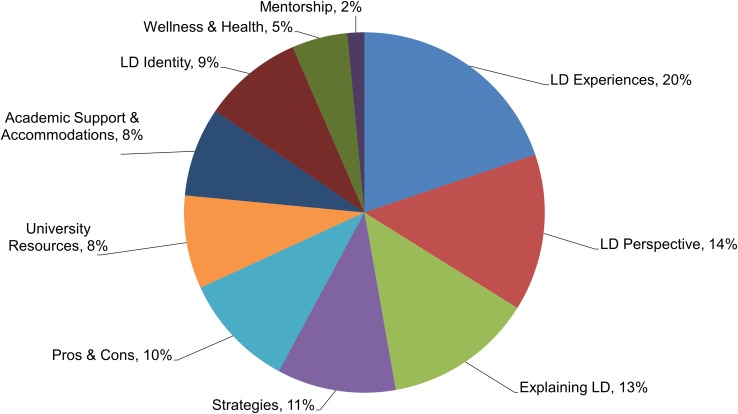
Topics included in undergraduates’ projects about learning disabilities (*n* = 102 projects, projects contained multiple topics).

The creation of student-led learning disability-awareness projects evolved from intended plans for the development of a student-led campus-wide learning disability-awareness event, which was not supported by participating undergraduates. We quickly learned that our undergraduates faced additional diagnostically related time constraints, such as slower reading and slower academic task completion, which prevented them from engaging in leadership of such time-intensive activities. Instead, undergraduates were willing to commit their time to the creation of individually led projects. The projects enabled the undergraduates to engage in a form of advocacy that they could self-manage on their own timeline while also enabling them to take leadership in directing the messaging used to address learning disability-related topics. Engagement in development of these advocacy projects were designed as a tool for encouraging the undergraduate’s self-esteem, self-acceptance, and comfort with promoting understanding of the disability in general. **Figure 3** depicts examples of undergraduates’ projects and **Figure 4** details topics included in the textual descriptions of the projects as provided or confirmed by the students. Thirty-five undergraduate participants (67%) submitted at least one project.

As a means of promoting undergraduates’ use and navigation of relevant disability-related services, undergraduates were also encouraged to meet with their disability office counselor at least once each semester. The disability office counselor meeting rate was 73%.

#### Interpersonal Level Implementation

Undergraduates met individually with their paired graduate student mentor at least once every other week in the fall and spring semesters for four consecutive non-summer semesters. The primary focus of the mentorship was to facilitate the undergraduate’s career development within the student’s chosen STEM field of study. On average, undergraduates met with their mentor in person 62% of expected meetings. An additional 19% of mentorship meetings were conducted via phone or video technologies (e.g., FaceTime and WhatsApp).

Mentor group meetings were held two to three times each semester to support mentors’ needs for understanding LDs and support mentor skill development, such as communication and coaching strategies and skills. Topical information regarding learning disabilities and universal design for learning were provided at the group meetings. Group meetings were also intended to serve as a forum for focused discussions and for answering mentors’ questions. However, as implementation of the CS^3^LD model evolved, the mentor group meetings also began to serve as a forum for the mentors to share experiences and strategies used in supporting students with LDs. If mentors were not able to attend a group mentor meeting, detailed meeting notes, discussion prompts, and topical information were electronically provided. Mentor group meetings were attended in-person at an average rate of 43% and via asynchronous means averaging 42%.

We originally envisioned the development of multi-disciplinary mentorship teams for each CS^3^LD undergraduate. Mentorship teams were to include the STEM graduate student/faculty dyad as the primary mentors and would also include other campus personnel who were identified by the undergraduate as important to his or her success (such as an advisor whom the student considered to be a supporter). However, the undergraduates expressed a preference for having only one primary mentor. Undergraduates expressed reluctance for direct mentorship support from the faculty mentor. Only a few ever met with their matched faculty mentor, and none reported meeting more than once. Additionally, graduate student mentors did not utilize the supplementary mentorship support from their paired STEM faculty mentor dyad member. Overall, expectations for the undergraduates to work with multiple mentors proved inefficient and impractical due to the undergraduates’ disability-related time constraints.

#### Institutional Level Implementation

The Partnership Council was the CS^3^LD model’s primary method for discussing, brainstorming, and addressing institutional level concerns regarding students with LDs. The Council was crucial for developing collaborations that facilitated efforts to raise awareness about LDs and principles of universal design for learning. Partnership Council meetings were held once each semester and evolved with only a few modifications. We originally envisioned that the Council would be composed of university administrators – those in a position to facilitate changes in campus policies. However, we quickly realized that in order to better foster changes in practice, we needed to incorporate both student service providers and student government representatives. Council meetings were initially co-led by the disability office director and the lead investigator who has disability training. Leadership responsibility was eventually assumed by the director of the campus disability office, which represented the institutionalization of the Council.

### Outcomes

#### Undergraduate Personal Level Outcomes

Undergraduates reported improvements in their self-efficacy as college students, their self-advocacy and communication about LDs, and their sense of connection with the campus community. As stated by one participant and reflective of his self-efficacy, “I have noticed that I have gotten a lot of good skills from coming to [the CS^3^LD group meetings], and my GPA has improved.” [U26] Some undergraduate participants attributed their academic success and their ability to graduate from college to the CS^3^LD supports received. As stated by one undergraduate,

“CS3LD changed my perspective on disabilities. It taught me to be comfortable in my own skin and to advocate for myself and others.… I will be graduating… Summa Cum Laude. I think this is all kind of funny. I did not think I would graduate when I was a freshman.… I cannot thank [CS3LD] enough for what [it has] done for me personally and academically…I hope to be like my mentors and enable others to be the best they can be.” [U22].

Two resounding themes, which are foundational to self-advocacy and communication about LDs, repeatedly emerged during the undergraduate group discussions. Firstly, undergraduate participants understood that having a learning disability and using their legally afforded classroom accommodations is not a “cop-out” or an excuse. As stated by one undergraduate, “Some people treat accommodations as an unfair advantage…when really it’s getting you to where everyone else is.”[U1].

Secondly, undergraduate participants were able to view their condition as a difference in thinking and learning rather than a deficiency or disability. “Because we have different ways of learning, it kind of makes us more open, or creative when we [are] approaching what we are doing in life or in school.” [U8] Some undergraduates were able to recognize strengths, such those in complex thinking, and could identify situations in which personal differences in their thinking and learning can be an asset. “I love complicated systems and figuring them out and making them efficient.” [U33].

Mentorship was an important source of connection to someone in their chosen STEM field. When asked about topics discussed with their mentors, one student responded “classes, research, physics, life, and politics.” [U4] For many, their mentor served as an important source of social support and encouragement. “It was a positive experience. [My mentor] helped me think in new ways and do things that I probably would not have done.” [U22] Others described developing friendships with their mentors and expressed intentions or desire to maintain a relationship. “[My mentor] was great. I just hope he’s still able to meet with me occasionally even though the commitment has ended.” [U11].

Quantitative indicators of CS^3^LD impacts from undergraduates’ surveys are summarized in **Tables 5**, **6**; **Table 5** delineates perceived impacts of CS3LD activities, and **Table 6** details changes in ratings specific to STEM professional development and self-advocacy. Undergraduate participants reported positive impacts from receipt of CS^3^LD supports (**Table 5**), with statistically significant improvements in several self-reported ratings of STEM professional development and self-advocacy (**Table 6**). ABCS scores from 35 undergraduates, and IIS and PGIS scores from 34 undergraduates were available for comparison following four semesters of CS^3^LD supports. Wilcoxon signed-rank test determined a statistically significant increase in ABCS score (*Mdn* = 0.380) after four semesters (*Mdn* = 3.790) compared to baseline scores (*Mdn* = 3.500), *z* = 3.252, *p* = 0.001. A significant increase in IIS score (*Mdn* = 0.426) was also found following four semesters (*Mdn* = 3.833) compared to baseline (*Mdn* = 3.310), *z* = 3.958, *p* < 0.001. PGIS scores did not significantly change.

**Table 5:** Perceived CS^3^LD Model impacts on undergraduate students with learning disabilities.

Area of perceived impact of CS^3^LD activities (*n*)	% Moderate or significant positive impact	% Minimal positive impact	% No impact	% Negative^∗^ impact	Mode response^α^
Understanding of learning disabilities (LD) (41)	78	20	2	0	Significant positive impact
Ability to articulate about LD in STEM (41)	78	20	2	0	Significant positive impact
Navigation of STEM field (42)	50	33	17	0	Moderate positive impact
Satisfaction with chosen STEM field (42)	57	21	21	0	Significant positive impact
Professional skills (42)	57	26	17	0	Moderate positive impact
Academic self-confidence (42)	62	24	14	0	Moderate positive impact
Connection to campus groups and supports (42)	52	40	7	0	Significant positive impact

^∗^*All negative response options are summarized as negative. ^α^Survey response options: Significant negative impact, moderate negative impact, minimal negative impact, no impact, minimal positive impact, moderate positive impact, significant positive impact*.

**Table 6:** Rating changes after four semesters for STEM professional development and self-advocacy within STEM field of study.

Question (*n*)	*Z* statistic	*p*-value^∗^ (2-tailed)	Median difference in ratings^α^
**Questions about STEM professional development**			
I make positive impressions with professional dress, conduct, and speech (34)	-3.119	**0.002**	0
I organize tasks and manage time to complete tasks by deadlines (34)	-2.025	0.043	+1
I respond promptly to phone calls, emails, or letters (34)	-3.073	**0.002**	+0.5
I am satisfied with my exposure to research and/or experiential opportunities in my STEM discipline at the University of Florida (34)	-2.488	0.013	+1
My University of Florida experiences have helped me identify personal strengths and weaknesses with my chosen STEM field (34)	-4.623	**<0.001**	+1
I have been assisted in identifying and overcoming my weaknesses within my STEM discipline (34)	-2.401	0.016	+1
**Questions about learning disability (LD) self-advocacy related to academics/STEM field of study**			
I know my strengths and limitations in the learning process (31)	-4.213	**<0.001**	+2
I know what accommodations I need to bypass my limitations (33)	-4.275	**<0.001**	+1
I can advocate for my specific LD needs with my instructors (33)	-4.094	**<0.001**	+1
I know about supports at University of Florida specific to LD students (32)	-3.88	**<0.001**	+1
I know how to be clear in requests and be prepared with explanations regarding my LD (33)	-3.716	**<0.001**	+1
I know how to communicate about my LD with others (33)	-3.654	**<0.001**	+1
I prepare ahead for communications about my LD with others (33)	-4.43	**<0.001**	+1
I am comfortable educating others about my LD (33)	-2.185	0.029	0
**Questions about learning disability (LD) self-advocacy related to health and wellbeing**			
My friends know about my LD and associated limitations (33)	-3.653	**<0.001**	+1
I can ask for help from my friends when faced with limitations (33)	-4.066	**<0.001**	+1
I have sought up-to-date information about my LD by talking to specialists and doing my own research (33)	-3.717	**<0.001**	+1
I understand my LD (33)	-4.351	**<0.001**	+1
I know how LD impacts academic progress and health Behaviors (11)	4.183	0.006	+2
I have an expert on LD on my healthcare team (33)	1.277	0.245	0

^∗^*Significance set at *p* < 0.003 using Bonferroni correction of *p* < 05/20. ^α^Response options: 5 = Strongly agree, 4 = Agree, 3 = Neutral, 2 = Disagree, 1 = Strongly disagree*.

#### Mentor Interpersonal Level Outcomes

Mentors increased their understanding about LDs and used this knowledge to support both their mentees and other students they worked with. The intended career paths of graduate student mentors varied and included intended careers in STEM education, industry, and research. Overwhelmingly, graduate student mentors reported greater appreciation of learning differences within their classrooms and within their research teams.

“Not only am I much more aware and sensitive to the number of students with LDs, but I have become proficient at recognizing symptoms, allowing me [to] change teaching styles if necessary.” [M94].

“I hold myself to a higher standard now when it comes to dealing with students and my [research assistants] more generally. Even something as simple as asking my [research assistants] if the way that I’m training them is working, and how they learn best.” [M109].

Mentors also reported gains in their own self-understanding as a result of their involvement in the CS^3^LD research project.

“I’ve become much more aware of how I learn and my own learning limitations, and being open about those so that I can open up a dialog with whoever I’m working with… In doing so, it has helped me avoid frustrating situations in all of my working relationships, not just those with LD.” [M109].

Another mentor, after gaining understanding of LDs, questioned his own learning challenges and was eventually diagnosed with a learning disorder.

Overall, the interpersonal level mentorship outcomes were positive. When graduate student mentors were surveyed, 33 of 38 respondents (87%) reported being very or extremely satisfied with their involvement in the CS^3^LD project. All respondents reported positive impacts in their understanding of LDs. “I feel that I am far more understanding and hopefully am able to provide guidance better now that I understand the difficulties associated with many of the LDs.” [M56].

#### Institutional Level Outcomes

At the institutional level, we aimed to change the culture of how students with LDs are supported on our campus. We did this through the creation of a campus-wide network of administrators, faculty, staff, and graduate students who are knowledgeable and coordinated in supporting the success of undergraduate students with LDs. For study investigators, mentors, and Partnership Council members, a gradual shift occurred in the viewing of LD as a learning difference instead of a health condition or a neurologic disorder.

Members of our Partnership Council identified and created opportunities for us to engage in already existing campus-based mechanisms for faculty and staff training. We used the words and advocacy messaging (i.e., learning disability awareness projects) created by undergraduates to illustrate learning disability-related experiences and to strengthen campus-based trainings. From the 102 learning disabilities-awareness projects created by undergraduate participants, 30 projects were selected for editing and readied for dissemination as public service information and/or training tools. Fourteen art works, five brochures/pamphlets, and eleven videos were disseminated with assistance from the Partnership Council. Disseminated messages covered topics such as the pros and cons of having a learning difference, issues faced by students with disabilities, communicating with professors, and raising awareness that individual with LDs live, study, and work among everyone.

Institutional-level impacts stemming from Partnership Council activities also resulted in enhanced coordination of student services. After undergraduate participants spoke of desires for learning disability-informed support in preparing to transition to the workplace, the University’s disability office and career services office partnered to create a series of workshops focusing on disability in the workplace. Additionally, the career services office partnered with the disability office to permanently house a career services advisor within the disability office. Student government involvement in the Partnership Council inspired one representative to spearhead the formation of a new student group. This group became a University sanctioned student group with a goal of empowering students with disabilities by promoting disability advocacy and leadership development opportunities for fostering a more inclusive campus community.

Following the Partnership Council’s institutional adoption, it was renamed as the Neurodiversity Council to reflect the evolved focus on supporting students with a broader range of learning styles and strengths. The range of cognitively diverse students on the University’s campus are supported by the Council’s focus on increasing campus members’ understanding of Universal Design for Learning and its application in classrooms, research laboratories, and across student-centered services such as student counseling, wellness services, and career advisement services.

Broader shifts in our campus’ understanding and valuation of diversity have also influenced the membership of and discussions within the Council. Specifically, disability is articulated as a type of diversity, which is valued for contributing to the richness of experiences and perspectives afforded from diverse campus members. As such, the Neurodiversity Council includes members from other diversity groups on campus. Disability is now included within broader campus activities and conversations regarding diversity.

### Social Validity

#### Campus Engagement

**FIGURE 5:**
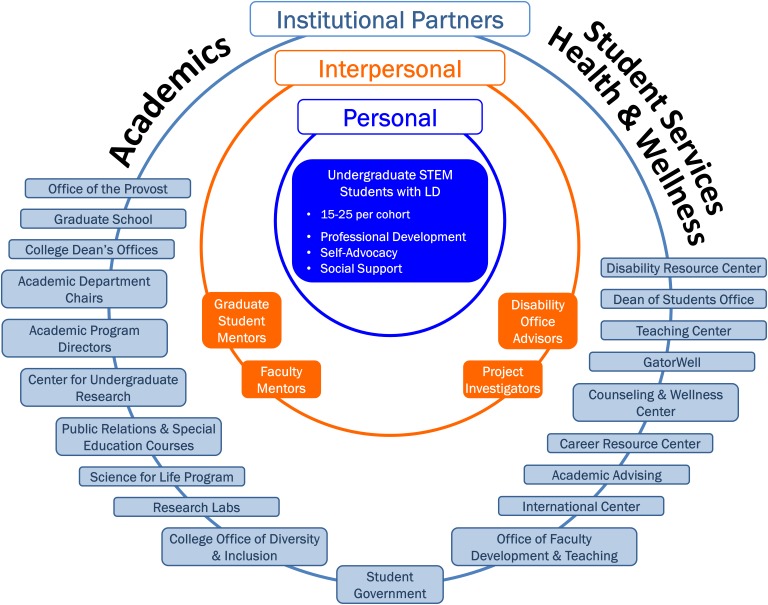
CS^3^LD campus engagement model for the University of Florida.

The social validity of the CS^3^LD model was evidenced by the high levels of interest during all phases of study recruitment and by participant retention at all levels. Response to recruitment efforts was largely enthusiastic with enrollment in CS^3^LD activities primarily limited by the study’s available resources. No difficulties were encountered in enrolling undergraduate, graduate student, and Partnership Council participants. The number of potential undergraduate and graduate student participants exceeded the targeted recruitment levels during the four cycles of undergraduate student recruitment and in three of four cycles of graduate student recruitment. Recruitment of the faculty mentors required the most effort. Continual expansion of Partnership Council membership occurred over the 4 years and was prompted by Council members’ discussions centering on strategies for improving awareness and learning disability-related supports. **Figure 5** details the campus engagement model at our university.

### Retention

Of the 52 undergraduates recruited, 46 were enrolled in time to participate for 2 years (i.e., four semesters) with 35 (76%) completing the 2 years. A total of 13 (25%) undergraduates withdrew from the study; six withdrew due to scheduling constraints, three left the university, two withdrew because of poor fit (e.g., did not like group meeting format), and two withdrew for positive opportunities (i.e., paid internship and early graduation). Of the 57 graduate student mentors, 42 (74%) completed mentorship obligations, 14 (24%) withdrew from the study, and 1 (2%) was never matched with an undergraduate mentee. Research participation was complicated for nine international students (i.e., eight graduate students and one undergraduate) by visa restrictions limiting compensation beyond the student’s educational program. Of these students, five graduate mentors and one undergraduate chose to participate in the study without compensation. No faculty mentors or Partnership Council members withdrew from project engagement without a change in employment (e.g., left or changed positions within the university, retired). Partnership Council members who left the Council facilitated their own replacement on the Council from alternate or incoming personnel within their unit.

## Discussion

This research tested the CS^3^LD model of campus-based holistic and integrated supports for students with LDs. Findings support the model’s strengths in advancing students’ self-efficacy, self-advocacy, self-management, STEM career exploration and professional development, and sense of campus connection. These findings are important as postsecondary success for students with disabilities is linked with receipt of non-academic supports, such as supports for building career awareness, self-advocacy, and interpersonal skills (Haber et al., 2016). Individual factors, such as those found to improve in our study, are important for supporting students’ academic satisfaction and success (VanZile-Tamsen, 2001; Pritchard and Wilson, 2003; Kim et al., 2010; Norvilitis and Reid, 2012; Showers and Kinsman, 2017). Study findings contribute evidence for the importance of providing holistic campus-based supports that are delivered in a purposeful and coordinated manner for students with learning and related disorders. Both this finding and the CS^3^LD model are consistent with contemporary conceptualizations of college success, which incorporate considerations of how well a student adjusts to college life and adapts to its demands within paradigms of success (Norvilitis and Reid, 2012),

The CS^3^LD model is distinctive in its coordinated provision of multi-level and holistic supports for students with LDs as they progress through college toward development of their desired careers and adult roles. On all levels, CS^3^LD model activities acknowledged and fostered understanding of challenges associated with LDs and focused on strengths. The CS^3^LD approach nurtured self-efficacy in the college student role while also promoting connections with supportive disability-informed others on campus. Supports nurtured students’ abilities for self-managing actions and societal expectations for independence and self-regulation that extended beyond the classroom to contexts of social activities, everyday life situations, and personal and professional role development.

For undergraduates in the study sample, positive changes were observed on measures of self-efficacy, campus integration, and self-advocacy. These findings contribute evidence that is indicative of the benefits in using a strengths-based approach when providing academic and psychosocial supports for transition-age individuals with developmental learning and related disorders. Personal and interpersonal level activities provided undergraduates with opportunities to interact with others in STEM with similar experiences and understanding of disability-related challenges and strengths. Such interpersonal experiences are important for nurturing personal and career identity development, persistence, and advanced scientific literacy (Lee and Fradd, 1998; Dika and D’Amico, 2016; Rahm and Moore, 2016). This study provides important insights into important processes which can be promoted by campus-based activities for cultivating the positive academic and psychosocial development of young adults with developmental learning disorders. As such, this study contributes knowledge salient to the fields of positive psychology (Peterson, 2006) and positive youth development (Lerner et al., 2005), disciplines which are believed to be important for lessening the damaging impacts of disability (Gable and Haidt, 2005).

At its core, the key model components of personal and interpersonal level activities were designed to promote the undergraduate’s self-regulation abilities. Self-regulation, in its role for supporting goal-directed behavior, is foundational for lifelong functioning and wellbeing (Murray et al., 2015). Importantly, interpersonal level activities extended promotion of undergraduates’ self-regulation by simultaneously developing the mentors’ capacities for co-regulation. Co-regulation within the CS^3^LD mentorship refers to the interactive process used by the mentors in supporting the regulation and development of their undergraduate mentee’s self-regulation abilities (Rosanbalm and Murray, 2017). Mentors’ co-regulation skills were bolstered through the provision of knowledge about LDs and support for mentoring skill and co-regulation strategy development provided within the mentor group meetings.

CS^3^LD personal and interpersonal activities are promising approaches for nurturing students’ self-regulation and for fostering the wellbeing of students with LDs. Several gaps exist within self-regulation intervention research; most notably a lack of young adult interventions, which include both self-regulation and co-regulation components, and evidence for relevant young adult functional outcomes of the self-regulation interventions (Murray and Rosanbalm, 2017). The positive changes observed in the sample’s social/campus integration, as measured with the IIS, suggest a potential relevant functional outcome for future self-regulation interventions of young adults with disabilities pursuing higher education. Additionally, this study contributes acutely needed empirically derived insights evidencing the promise of including co-regulation skill development (i.e., learning disability-informed mentorship) within interventions for self-regulation (i.e., group meetings with engagement in a tangible self-advocacy activity) as developed for young adult populations.

Undergraduates preferred mentorship from a more senior student with experience in navigating a path comparable to the one desired by the undergraduate mentee. This aspect of the mentorship, in combination with the mentor’s understanding of LDs, represent the key aspects of the mentorship which facilitated the mentee’s ability to identify with his or her mentor. This is consistent with mentorship research reporting higher quality learning and better quality mentor-mentee relationships with mentors who are perceived as similar (Allen and Eby, 2003). This finding is important for guiding potential implementation of the CS^3^LD model on college campuses without or with smaller graduate programs.

CS^3^LD model is an interdisciplinary approach for holistically supporting student success on college campuses. The incorporation of multiple disciplines within the research team and among all levels of research participants was strength of this study. The wide-ranging disciplinary perspectives served to provide diverse perspectives that bolstered the continuous improvement process used in implementing and refining model activities. The study team was represented by investigators from engineering, life sciences, psychology, health education, and rehabilitation. These varying disciplinary perspectives strengthened the study design, implementation, and interpretation of research findings.

Our combined use of continuous improvement and participatory action research approaches was integral to decisions made in refining and implementing CS^3^LD model activities. Incorporating the voices of the students with LDs alongside mentor voices was critical in informing institutional level activities and served to strengthen campus-based trainings regarding implementation of universal design for learning created for advisors and instructors. Institutional level activities were aimed at creating a supportive campus environment for students with learning and other disabilities. Shifting the composition of the Partnership Council to include membership of non-administrators helped focus institutional level efforts from potential policy changes to actions for changes in service delivery. Changes resulting from efforts of Partnership Council members had an immediate and direct impact on service delivery/supports for students with LDs. Involvement of representatives from student government and other diversity (e.g., gender diversity and ethnic minority) groups within the Council resulted in framing LDs and disability in general, as a matter of diversity and inclusion.

This study illustrates the use of campus-based processes that were instrumental in facilitating positive changes in culture and practice. The concept of neurodiversity as adopted by the university-wide Council highlights the fact that individuals with LDs, and many other invisible disabilities, contribute diverse experiences and skillsets that serve to enrich the campus environment. Incorporating disability into postsecondary educational institutions’ diversity efforts is a key approach for impacting institutional changes. CS^3^LD model activities are important for their potential to raise awareness and shape social-cultural normative attitudes about disability on college campuses while simultaneously fostering the individual level success of academically and psychosocially vulnerable students.

### Limitations

The primary focus of this research was to test the implementation of the CS^3^LD model of support whereby the means in which the model was implemented were specific to our large research intensive university. Importantly, this study was not designed to test the effectiveness of the interventions implemented, but rather to test the implementation of the model. While overarching lessons can be learned from this implementation, care should be exercised in attempting to generalize implementation strategies described. Future studies should test model implementation at other universities, with other clinical groups, and with students not engaged in a STEM education. Additional research is needed that enables a statistical multi-level analysis in order to more robustly test the multi-level CS^3^LD model. Additionally, judicious interpretations of personal level (i.e., undergraduate participant) outcomes should be used as this was a one-group pre-post analysis of data from a small sample of students with LDs. Further investigation is needed to test for treatment effects of the psychoeducational groups and mentorship interventions used within the CS^3^LD model.

## Conclusion

The CS^3^LD model, as a framework for coordinated holistic campus-based interventions, is a promising practice that positively impacted participating students with LDs, mentors, and the campus environment. Implementation of a comprehensive, multi-level, and coordinated model of supports for students with LDs is feasible and was well accepted by students and campus stakeholders for fostering the performance, development, and campus connections of undergraduates with LDs. Mentorship from someone in a similar field of study and who understands LDs as differences in learning, is a key component of the model for better promoting the undergraduates’ sense of self-confidence, success, and connectedness. Transforming the understanding of LDs as differences in learning for individuals working and learning on college campuses can be a powerful strategy for harnessing the diverse strengths of students aspiring to join the STEM professional workforce. Multi-level and holistic supports are important for assisting young people in meeting the multifaceted demands associated with striving for college success as an individual with a developmental learning disorder. The continued development and testing of holistic strengths-based interventions that advance the self-regulation abilities of young adult college students with LDs is merited. The proliferation of interdisciplinary, multi-level, and holistic supports is warranted for promoting the wellbeing of students with learning and related disorders and for improving the postsecondary educational opportunities and experiences for these individuals.

## Ethics Statement

This study was carried out in accordance with the recommendations of Human Subjects Protection Guidelines, University of Florida Gainesville Health Science Center Institutional Review Board (IRB-01). The protocol was approved by the University of Florida IRB-01. All subjects gave written or oral informed consent, as appropriate, in accordance with the Declaration of Helsinki.

## Data Availability Statement

The datasets generated and/or analyzed during the current study are available from the corresponding author upon reasonable request.

## Author Contributions

All authors contributed extensively to the work presented in this paper. CK, C-YW, M-FL, SP, CB, AD, and WM contributed to the study conception and design. SM and DS contributed to acquisition of the data. CK, C-YW, M-FL, SP, CB, and DS contributed to analysis and interpretation of the data. CK, SM, M-FL, and C-YW drafted the work and all authors contributed critical appraisal and revision for important intellectual content. All authors provide final approval of the version submitted for publication and agree to be accountable for all aspects of the work as presented.

## Conflict of Interest Statement

The authors declare that the research was conducted in the absence of any commercial or financial relationships that could be construed as a potential conflict of interest.

## References

[B1] Allen T. D. Eby L. T. (2003). Relationship effectiveness for mentors: factors associated with learning and quality. *J. Manag.* 29 469–486. 10.1016/s0149-2063_03_00021-7

[B2] American Psychiatric Association (2013). *Diagnostic and Statistical Manual of Mental Disorders.* Washington, DC: American Psychiatric Association 10.1176/appi.books.9780890425596

[B3] Asghar A. Sladeczek I. E. Mercier J. Beaudoin E. (2017). Learning in science, technology, engineering, and mathematics: supporting students with learning disabilities. *Can. Psychol.* 58 238–249. 10.1037/cap0000111

[B4] Bandura A. (1986). *Social Foundations of Thought and Action: A Social Cognitive Theory*. Englewood Cliffs, NJ: Prentice-Hall, Inc.

[B5] Bandura A. (1998). Health promotion from the perspective of social cognitive theory. *Psychol. Health* 13 623–649. 10.1080/08870449808407422

[B6] Bandura A. (2005). “The evolution of social cognitive theory,” in *Great Minds in Management*, eds Smith K. G. Hitt M. A. (Oxford: Oxford University Press), 9–35.

[B7] Baum C. M. Law M. (1997). Occupational therapy practice: focusing on occupational performance. *Am. J. Occup. Ther.* 51 277–288. 10.5014/ajot.51.4.277 9085726

[B8] Brigham F. J. Scruggs T. E. Mastropieri M. A. (2011). Science education and students with learning disabilities. *Learn. Disabil. Res. Pract.* 26 223–232. 10.1111/j.1540-5826.2011.00343.x

[B9] Burgstahler S. E. (2008). “Universal design of instruction: from principles to practice,” in *Universal Design in Higher Education: From Principles to Practice*, eds Burgstahler S. E. Cory R. C. (Cambridge, MA: Harvard Education Press), 23–43.

[B10] Chen X. (2013). *STEM Attrition: College Students’ Paths Into and Out of STEM Fields (NCES 2014-001)*. Washington, DC: National Center for Education Statistics.

[B11] Chen X. Ho P. (2012). *STEM in Postsecondary Education: Entrance, Attrition, and Coursetaking Among 2003-04 Beginning Postsecondary Students (NCES 2013-152)*. Washington, DC: National Center for Education Statistics.

[B12] Cortiella C. Horowitz S. H. (2014). *The State of Learning Disabilities: Facts, Trends and Emerging Issues*, 3rd Edn, New York, NY: National Center for Learning Disabilities.

[B13] Dika S. L. D’Amico M. M. (2016). Early experiences and integration in the persistence of first-generation college students in STEM and non-STEM majors. *J. Res. Sci. Teach.* 53 368–383. 10.1002/tea.21301

[B14] Gable S. L. Haidt J. (2005). What (and why) is positive psychology? *Rev. Gen. Psychol.* 9 103–110. 10.1037/1089-2680.9.2.103

[B15] Haber M. G. Mazzotti V. L. Mustian A. L. Rowe D. A. Bartholomew A. L. Test D. W. (2016). What works, when, for whom, and with whom: a meta-analytic review of predictors of postsecondary success for students with disabilities. *Rev. Educ. Res.* 86 123–162. 10.3102/0034654315583135

[B16] Hain L. A. Hale J. B. Kendorksi J. G. (2009). “The comorbidity of psychopathology in cognitive and academic SLD subtypes,” in *Emotional Disorders: A Neuropsychological, Pychopharmacological, and Educational Perspective*, eds Pfeifer S. G. Rattan G. (Middletown, CT: School Neuropsychology Press), 199–226.

[B17] Johnson E. S. Humphrey M. Mellard D. F. Woods K. Swanson H. L. (2010). Cognitive processing deficits and students with specific learning disabilities: a selective meta-analysis of the literature. *Learn. Disabil. Q.* 33 3–18. 10.1177/073194871003300101

[B18] Kim E. Newton F. B. Downey R. G. Benton S. L. (2010). Personal factors impacting college student success: constructing College Learning Effectiveness Inventory (CLEI). *Coll. Stud. J.* 44 112–125.

[B19] Kreider C. M. Bendixen R. M. Lutz B. J. (2015). Holistic needs of university students with invisible disabilities: a qualitative study. *Phys. Occup. Ther. Pediatr.* 35 426–441. 10.3109/01942638.2015.1020407 25978113 PMC4636462

[B20] Lahmers A. G. Zulauf C. R. (2000). Factors associated with academic time use and academic performance of college students: a recursive approach. *J. Coll. Stud. Dev.* 41 544–556.

[B21] Lee O. Fradd S. H. (1998). Science for all, including students from non-English-language backgrounds. *Educ. Res.* 27 12–21. 10.2307/1176619

[B22] Lerner R. M. Almerigi J. B. Theokas C. Lerner J. V. (2005). Positive youth development: a view of the issues. *J. Early Adolesc.* 25 10–16. 10.1177/0272431604273211

[B23] Lorig K. R. Holman H. R. (2003). Self-management education: history, definition, outcomes, and mechanisms. *Ann. Behav. Med.* 26 1–7. 10.1207/s15324796abm2601_01 12867348

[B24] Macan T. H. Shahani C. Dipboye R. L. Phillips A. P. (1990). College students’ time management: correlations with academic performance and stress. *J. Educ. Psychol.* 82 760–768. 10.1186/s13104-017-2439-6 28253905 PMC5335855

[B25] MacDonald C. (2012). Understanding participatory action research: a qualitative research methodology option. *Can. J. Action Res.* 13 34–50. 10.1016/j.midw.2012.07.006 22901497

[B26] Moll K. Göbel S. M. Gooch D. Landerl K. Snowling M. J. (2016). Cognitive risk factors for specific learning disorder: processing speed, temporal processing, and working memory. *J. Learn. Disabil.* 48 272–281. 10.1177/0022219414547221 25124507

[B27] Murray D. W. Rosanbalm K. (2017). *Current Gaps and Future Directions for Self-Regulation Intervention Research: A Research Brief*. OPRE Report No. 2017-93 Washington, DC: Office of Planning, Research, and Evaluation.

[B28] Murray D. W. Rosanbalm K. Christopoulos C. (2016). *Self Regulation and Toxic Stress: Seven Key Principles of Self Regulation in Context*. OPRE Report No. 2016-39 Washington, DC: Office of Planning, Research, and Evaluation.

[B29] Murray D. W. Rosanbalm K. Christopoulos C. Hamoudi A. (2015). *Self-Regulation and Toxic Stress: Foundations for Understanding Self-Regulation from an Applied Developmental Perspective*. OPRE Report No. 2015-21 Washington, DC: Office of Planning, Research and Evaluation.

[B30] National Science, and Board (2014). *Science and Engineering Indicators 2014 (NSB 14-01)*. Arlington, TX: National Science Foundation.

[B31] Newman L. Wagner M. Knokey A.-M. Marder C. Nagle K. Shaver D. (2011). *The Post-High School Outcomes of Young Adults With Disabilities up to 8 Years After High School. A Report From the National Longitudinal Transition Study-2 (NLTS2)*. Menlo Park, CA: SRI International.

[B32] Norvilitis J. M. Reid H. M. (2012). Predictors of academic and social success and psychological well-being in college students. *Educ. Res. Int.* 2012:6 10.1155/2012/272030

[B33] Pascarella E. T. Terenzini P. T. (1980). Predicting freshman persistence and voluntary dropout decisions from a theoretical model. *J. High. Educ.* 51 60–75. 10.2307/1981125

[B34] Peters D. H. Adam T. Alonge O. Agyepong I. A. Tran N. (2013). Implementation research: what it is and how to do it. *BMJ* 347:f6753. 10.1136/bmj.f6753 24259324

[B35] Peterson C. (2006). *A Primer in Positive Psychology.* Oxford: Oxford University Press.

[B36] Pritchard M. E. Wilson G. S. (2003). Using emotional and social factors to predict student success. *J. Coll. Stud. Dev.* 44 18–28. 10.1353/csd.2003.0008

[B37] Rahm J. Moore J. C. (2016). A case study of long-term engagement and identity-in-practice: insights into the STEM pathways of four underrepresented youths. *J. Res. Sci. Teach.* 53 768–801. 10.1002/tea.21268

[B38] Richards L. (2015). *Handling Qualitative Data: A Practical Guide*. Thousand Oaks, CA: Sage Publications, Inc.

[B39] Richards L. Morse J. (2013). *README FIRST for a User’s Guide to Qualitative Methods*. Thousand Oaks, CA: Sage Publications, Inc.

[B40] Robitschek C. (1998). Personal growth initiative: the construct and its measure. *Meas. Eval. Couns. Dev.* 30 183–198. 10.1177/1073191114524019 24569534

[B41] Rosanbalm K. Murray D. W. (2017). *Caregiver Co-Regulation Across Development: A Practice Brief*. OPRE Brief Report No. 2017-80 Washington, DC: Office of Planning, Research.

[B42] Sander P. Sanders L. (2003). Measuring confidence in academic study: a summary report. *J. Res. Educ. Psychol. Psychopedag.* 1 113–130.

[B43] Sander P. Sanders L. (2006). Understanding academic confidence. *Psychol. Teach. Rev.* 12 29–42.

[B44] Scanlon D. (2013). Specific learning disability and its newest definition: which is comprehensive? and which is insufficient? *J. Learn. Disabil.* 46 26–33. 10.1177/0022219412464342 23144061

[B45] Sharfi K. Rosenblum S. (2014). Activity and participation characteristics of adults with learning disabilities - a systematic review. *PLoS One* 9:e106657. 10.1371/journal.pone.0106657 25184315 PMC4153678

[B46] Showers A. H. Kinsman J. W. (2017). Factors that contribute to college success for students with learning disabilities. *Learn. Disabil. Q.* 40 81–90. 10.1177/0731948717690115

[B47] Smith-Spark J. H. Henry L. A. Messer D. J. Edvardsdottir E. Ziêcik A. P. (2016). Executive functions in adults with developmental dyslexia. *Res. Dev. Disabil.* 5 323–341. 10.1016/j.ridd.2016.03.001 26970859

[B48] Tanner K. (2009). Adult dyslexia and the ‘conundrum of failure’. *Disabil. Soc.* 24 785–797. 10.1080/09687590903160274

[B49] Trainin G. Swanson H. L. (2005). Cognition, metacognition, and achievement of college students with learning disabilities. *Learn. Disabil. Q.* 28 261–272. 10.2307/4126965

[B50] VanZile-Tamsen C. (2001). The predictive power of expectancy of success and task value for college students’ self-regulated strategy use. *J. Coll. Stud. Dev.* 42 233–241.

[B51] World Health Organization (2007). *International Classification of Functioning, Disability and Health: Children & Youth Version ICF-CY*. Geneva: World Health Organization.

